# Evaluation of the diagnostic sensitivity and specificity of two pen-side tests for detecting African swine fever virus in experimentally infected pigs

**DOI:** 10.1007/s00705-024-06098-0

**Published:** 2024-07-30

**Authors:** Hanh D. Vu, Hung Q. Luong, Huong T.L. Lai, Hoa T. Nguyen, Trang H. Pham, Lam Q. Truong, Giap V. Nguyen, Hiep L.X. Vu

**Affiliations:** 1 Vietnam National University of Agriculture, Hanoi, 100000 Vietnam; 2 Nebraska Center for Virology, School of Veterinary Medicine and Biomedical Sciences, University of Nebraska-Lincoln, Lincoln, NE 68583 USA; 3 Department of Animal Science, Nebraska Center for Virology, University of Nebraska-Lincoln, Lincoln, NE 68583 USA

## Abstract

African swine fever virus (ASFV) has spread through many countries and regions worldwide, causing significant losses. Timely detection of ASFV-infected pigs is crucial for disease control. In this study, we assessed the performance of two pen-side tests: a portable real-time PCR (qPCR) test for detecting viral genomic DNA and a lateral flow immunoassay (LFIA) for detecting viral antigens. To determine the time from infection to the earliest detection, 10 ASFV-seronegative pigs were inoculated intramuscularly with 10^4.0^ hemadsorption dose 50 of a highly virulent ASFV strain. Whole blood and oral swab samples were alternately collected from each group of five pigs daily until all succumbed to the infection. Samples were promptly subjected to the two pen-side tests upon collection, and a subset was transported to a veterinary diagnostic laboratory for analysis using a reference qPCR assay. Viral genomic DNA was consistently detected by the reference qPCR assay in all blood samples from 2 days postinfection (dpi), preceding the onset of clinical signs, and in oral swabs from 4 dpi onwards. The portable qPCR test demonstrated comparable performance to the reference qPCR assay for both whole blood and oral swab samples. The LFIA exhibited 100% specificity when testing with whole blood samples but showed reduced sensitivity, particularly for blood samples collected early or late after infection. The antigen test did not perform well with oral swabs.

**Supplementary Information:**

The online version contains supplementary material available at 10.1007/s00705-024-06098-0.

## Introduction

African swine fever virus (ASFV) is the etiological agent of African swine fever (ASF), a severe disease in pigs with a case-fatality rate approaching 100% [9]. ASF was first described in the early 1900s in Kenya and has since become endemic in most sub-Saharan countries in Africa [20]. The virus emerged in Portugal in 1957 and quickly spread to other European countries, South America, and the Caribbean. The disease was eradicated from countries outside Africa in the mid-1990s, except in Sardinia, Italy [21]. In 2007, a highly virulent ASFV strain emerged in Georgia and spread through Russia and Eastern Europe [5]. In 2018, the virus emerged in China and quickly spread through many Asian countries [31]. According to the World Organization for Animal Health (WOAH, formerly O.I.E.), ASF cases have been reported in 89 countries across Africa, Europe, Asia, Oceania, and Central America since 2005 [28]. The socio-economic impact of ASF is enormous. Within one year after ASF was first reported, China’s hog inventory was down by 41.4%, and the size of breeding herds declined by 38.9% [4].

The Vietnamese government recently approved the commercial use of two live-attenuated ASFV vaccines in domestic pigs. One vaccine, AVAC ASFV LIVE, produced by AVAC Vietnam Joint Stock Company, was developed using a recombinant ASFV strain containing a deletion of six genes from a multigene family [18]. The other vaccine, NAVET-ASFVAC, produced by Navetco Central Veterinary Medicine Company, was developed using a recombinant ASFV strain with a deletion of the I177L gene [2]. The safety and efficacy data for these two vaccines are not officially available. Currently, these vaccines are used cautiously in Vietnam due to concerns about potential reversion to virulence. The WOAH has recently raised concerns that the use of non-compliant and poor-quality vaccines may result in recombination between the vaccine virus and field virus, leading to novel viral strains that are capable of causing acute, chronic, or persistent infections [29].

The primary measure to control ASFV when it emerges in new countries or regions relies on stringent biosecurity practices involving movement restrictions, quarantine, and depopulation of affected herds to prevent viral transmission. However, a partial depopulation approach has been proposed in endemic areas, where only infected animals are selectively culled [7]. An initial study suggested that this approach could save an average of 58% of pigs on affected farms, allowing them to remain in production and thereby mitigating the impact on the economy and food security [17]. In this context, timely detection of ASFV-infected pigs is crucial for effective ASFV control [22]. Formal diagnosis of ASFV must be conducted in approved animal health laboratories. As such, samples must be transported to these laboratories, causing delays from sample collection to test results. Reliable pen-side tests are needed to detect ASFV infections directly in the field. Although these test results might not receive official recognition, they enable the immediate implementation of control measures, such as restricting animal movement, until results are confirmed by reference laboratories [23]. Consequently, pen-side tests could be valuable tools for ASF control programs.

Several types of pen-side tests for detecting ASFV-infected pigs have been developed. They are broadly classified into two groups. One group relies on a lateral flow immune assay (LFIA) to detect a viral antigen. The primary targets for LFIA development are the viral proteins p72 and p32 (also known as p30) [12, 15, 23, 32]. The other group utilizes PCR to detect viral genomic DNA. Two different approaches are used to develop a pen-side PCR. In one case, isothermal DNA amplification technologies are employed to amplify the viral genomic DNA, eliminating the need for expensive thermocyclers and thus allowing on-site testing [13, 26, 27, 30]. In another case, PCR reagents are freeze-dried to enable storage at ambient temperature, and the PCR is run on a portable, battery-powered thermal cycler [14, 33].

In this study, we evaluated the diagnostic performance of two pen-side tests: the LFIA designed for detecting viral antigens and the portable qPCR test designed for detecting viral genomic DNA.

## Materials and methods

### Animal experiments and sample collection

Ten six-week-old, ASFV-negative pigs were obtained from a local farm in Vietnam and housed in an isolated room at the Vietnam National University of Agriculture (VNUA) animal research facility. Following a week of acclimatization, whole blood and oral swabs were collected from each pig on days − 7, −4, −1, and 0 of the study. These pre-study samples (ASFV negative) were utilized to determine the specificity of the tests.

The virulent virus ASFV used in this study was isolated from the spleen of a domestic pig in an ASF outbreak in northern Vietnam in 2020. It was propagated for four passages and titrated in porcine alveolar macrophages [25]. Pigs were inoculated intramuscularly with 1 mL of inoculum containing 10^4.0^ hemadsorption dose 50 (HAD_50_) of the ASFV strain. Whole blood samples with anticoagulant and oral swabs were collected daily for 10 days. To prevent sampling the same pigs on two consecutive days, which could affect their well-being, these 10 pigs were ear-tagged from 1 to 10. Pigs with an odd ear-tag number were sampled on odd dates, and those with an even ear-tag number were sampled on even dates. This sampling scheme allowed us to obtain daily samples after inoculation to determine the time until the earliest detection of ASF in the experimentally infected pigs.

Each sample was aliquoted into two portions. One portion was analyzed immediately after collection with the two pen-side tests. The other portion was transported to the laboratory and stored at −80°C until the end of the experiment, at which point it was tested using the reference real-time PCR assay.

The animals were monitored daily by an appointed veterinarian. Pigs that reached humane endpoints were euthanized, and a necropsy was performed. The appointed veterinarian determined the humane endpoints, following the clinical evaluation scoring system, including fever, body condition, behavior, and digestive and respiratory signs, as described previously [10].

### Lateral flow immunoassay

The lateral flow immunoassay (LFIA) INgezim ASF CROM Ag, 11.ASF.K42 was obtained from Ingenasa (Madrid, Spain) [23]. Whole blood and oral swab samples were subjected to LFIA testing following the manufacturer’s protocol. In brief, 20 µL of the test sample was added to the sample window. One minute later, 3 to 4 drops of running buffer were applied to the sample window, and test outcomes were interpreted after 10 minutes. Positive samples displayed two colored lines at positions C and T, while negative samples displayed a single-colored line at the C position. If no colored line appeared, the test was deemed invalid and was repeated.

### Portable qPCR

The dried qPCR reagents, ASFV 3.0 with Inhibition Control, which contained primers, probes, buffer, and enzyme optimized for the detection of ASFV genomic DNA, along with the portable PCR instrument, T-COR8, were acquired from Tetracore Inc. (Rockville, Maryland, USA). The assay was performed strictly according to the manufacturer’s manual. Briefly, whole blood and oral swab samples were diluted 40-fold in nuclease-free, molecular-grade water. Subsequently, 5 µL of the diluted sample was dispensed into a T-COR8 tube containing 20 µL of rehydrated ASFV 3.0 reagents. The reaction was run on a T-COR8 real-time PCR instrument with the thermal cycling protocol comprising one cycle at 95°C for 3 minutes, followed by 45 cycles at 95°C for 10 seconds and 60°C for 30 seconds. Samples were considered positive if the cycle threshold (Ct) values were equal to or below 38, as indicated by the manufacturer. The portable qPCR, according to the manufacturer’s specifications, has a detection limit of 13 copies per PCR reaction.

### Reference real-time PCR

DNA was extracted using a MagMAX Viral/Pathogen Nucleic Acid Isolation Kit (Applied Biosystems) in a King Fisher Duo Prime DNA/RNA automatic extraction system. ASFV genomic DNA was quantified using a real-time PCR assay with primers and probes specific for the viral p72 genes [24] and Platinum Quantitative PCR SuperMix-UDG (Invitrogen). The PCR was performed on CFX Opus 96 instruments (Bio-Rad) with a thermal cycling protocol comprising one cycle of 95°C for 2 minutes, followed by 45 cycles at 95°C for 15 seconds and 60°C for 45 seconds. Samples were considered positive if the Ct values were equal to or below 38. The limit of detection for the reference real-time PCR assay was defined as 5.7–57 copies of the ASFV genome [24].

### Data analysis

The sensitivity and specificity of the two pen-side tests were calculated relative to the reference qPCR test results, following a procedure described previously [1]. Variables measured included true positives (TP), true negatives (TN), false positives (FP), and false negatives (FN). Sensitivity was calculated as 100 × TP / (TP + FN), while specificity was calculated as 100 × TN / (TN + FP).

## Results

### Clinical observations and mortality

**Fig. 1:**
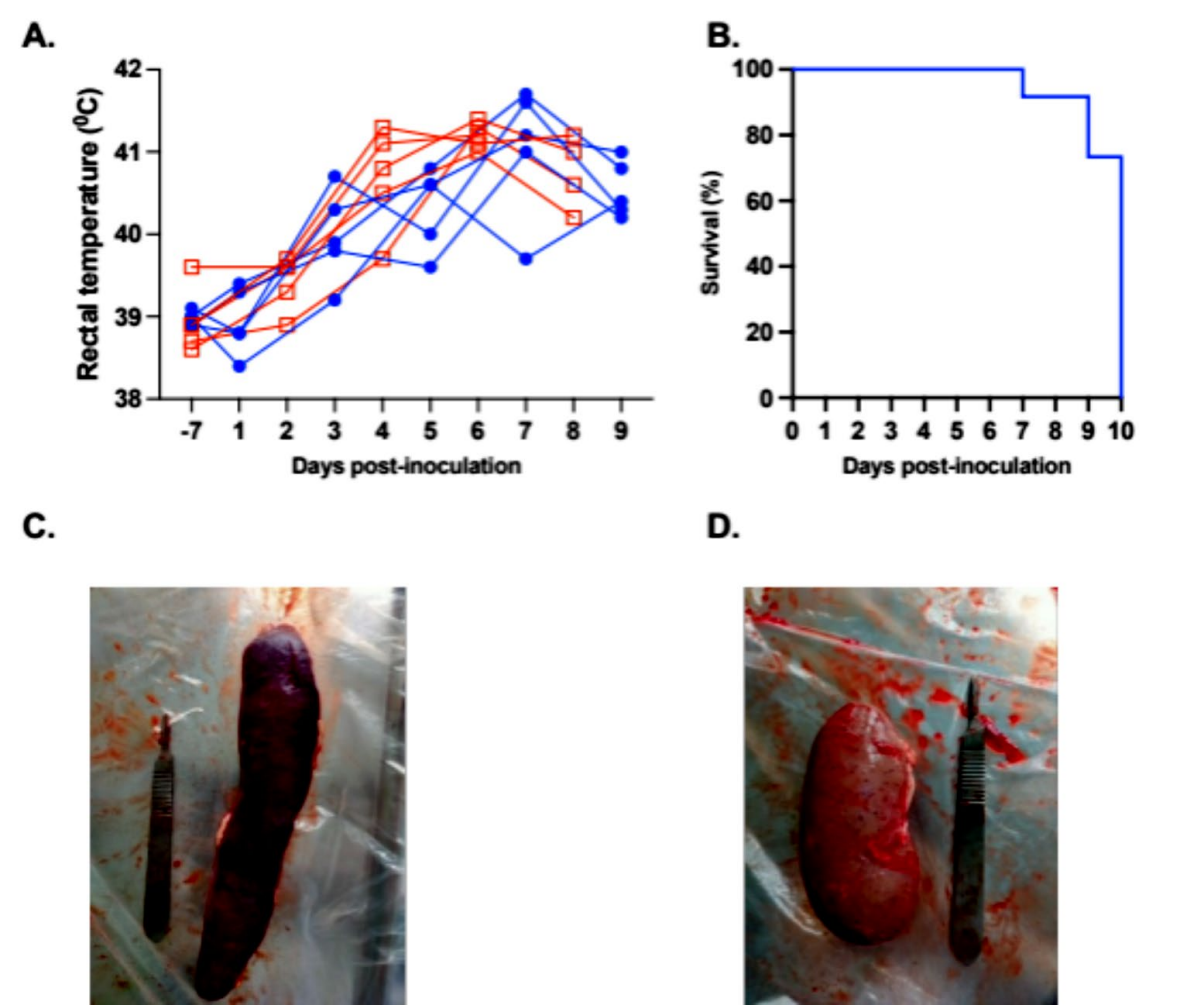
(**A**) Rectal temperature. Blue and red lines depict data collected from pigs on odd and even days postinfection, respectively. (**B**) Survival rate. (**C** and **D**) Representative photos of spleen and kidney taken during necropsy

We first observed clinical signs, such as fever (Fig. 1A), loss of appetite, and skin hemorrhages at 4 days postinfection (dpi). One pig reached the humane endpoint and was euthanized at 7 dpi, two pigs were euthanized at 9 dpi, and the remaining seven pigs were euthanized at 10 dpi (Fig. 1B). During necropsy, the pigs displayed typical ASF lesions, including an enlarged spleen and hemorrhages on serous surfaces, such as the kidney capsules and lymph nodes (Fig. 1C and D).

### Test results with whole blood samples

**Fig. 2:**
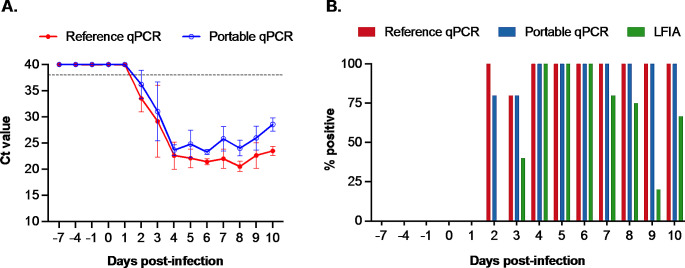
Test results obtained using whole blood samples. (**A**) Cycle threshold (Ct) values obtained using the reference and portable qPCR tests. The horizontal dashed line at a Ct value 38 indicates the assay cutoff. Samples with Ct values below this threshold were deemed positive. (**B**) The percentage of samples testing positive on each sampling day

We evaluated test performance using whole blood samples. The reference qPCR test detected viral genomic DNA in all five blood samples collected at 2 dpi, with a mean Ct value around 33.5. The Ct value decreased rapidly by 4 dpi, indicating a sharp increase in the viral load in the blood (Fig. 2A). The portable qPCR exhibited a Ct pattern comparable to the reference qPCR.

Regarding the detection rate, the reference qPCR identified ASFV genomic DNA in all blood samples collected from 2 dpi onward, except at 3 dpi, when it detected viral DNA in 4 out of 5 samples (80%) (Fig. 2B). The portable qPCR also detected viral genomic DNA starting from 2 dpi, with 80% (4 out of 5) of samples testing positive, and its detection rate increased to 100% at 4 dpi, remaining consistent until the end of the study. The LFIA detected viral antigens in samples collected from 3 dpi, one day later than the reference and portable qPCR. The LFIA consistently identified antigens in all blood samples collected between 4 and 6 dpi, with a subsequent decrease in the detection rate observed for samples collected from 7 dpi onward (Fig. 2B).

In this study, we analyzed 82 whole blood samples: 35 collected before and 47 collected after inoculation. Of these 82 samples, 41 tested positive and 41 tested negative using the reference qPCR assay (Tables 1 and 2). Both pen-side kits accurately identified all 41 samples that tested negative using the reference qPCR assay. The portable qPCR test detected 40 out of 41 (97.6%) samples that tested positive using the reference real-time PCR assay (Table 1). The LFIA identified 27 out of 41 (65.9%) samples that tested positive using the reference qPCR assay (Table 2).

**Table 1:** Comparison of portable qPCR and reference qPCR test results for whole blood samples

Portable qPCR	Reference qPCR		Total
	Positive	Negative	
Positive	40	0	40
Negative	1	41	42
Total	41	41	82

Sensitivity: 97.6% Specificity: 100%

**Table 2:** Comparison of LFIA and reference qPCR test results for whole blood samples

LFIA	Reference qPCR		Total
	Positive	Negative	
Positive	27	0	27
Negative	14	41	55
Total	41	41	82

Sensitivity: 65.9% Specificity: 100%

Among the 14 positive samples that the LFIA failed to detect, seven were collected early (2 and 3 dpi), and seven were collected late (7 dpi onward) in the course of infection (Supplementary Table S1). The early samples had Ct values ranging from 26.17 to 36.5, suggesting moderate to low viral loads (Supplementary Table S1). Conversely, the late samples had Ct values ranging between 20.59 and 26.83, indicating higher viral loads than the early samples. While the LFIA’s inability to detect samples collected early in the course of infection might be attributed to low viral loads, this cannot explain its failure to detect samples collected later in the course of infection.

### Test results with oral swabs

**Fig. 3:**
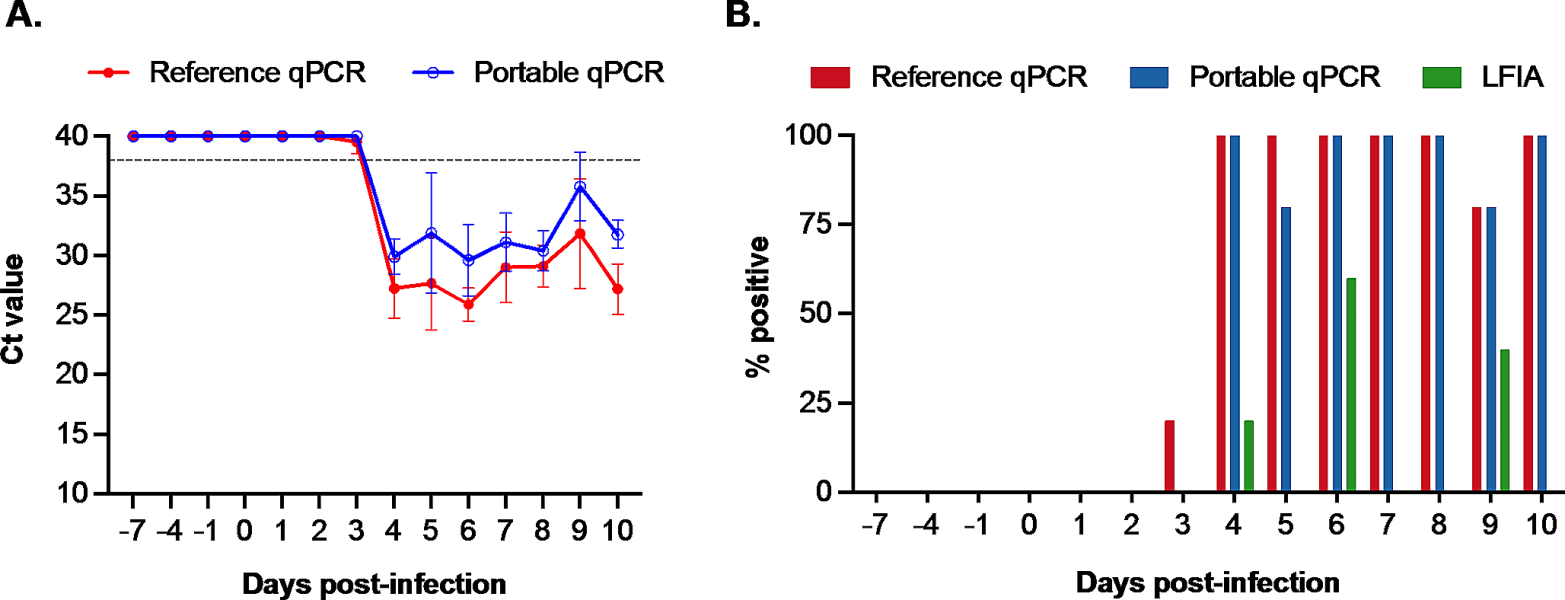
Test results obtained using oral swabs. (**A**) Cycle threshold (Ct) values obtained using the reference and portable qPCR tests. The horizontal dashed line at a Ct value 38 indicates the assay cutoff. Samples with Ct values below this threshold were deemed positive. (**B**) The percentage of samples testing positive on each sampling day

All pigs that were experimentally inoculated with a virulent ASFV strain shed the virus in their oral secretions, as indicated by the consistent detection of viral genomic DNA in all oral swabs starting from 4 dpi by the reference qPCR assay. We did not observe significant differences in the Ct patterns between the reference and portable real-time PCR tests (Fig. 3A).

The reference qPCR assay detected viral genomic DNA in 1 of 5 (20%) oral swabs collected at 3 dpi and in all swabs collected at 4 dpi or later (Fig. 3B). The portable qPCR test exhibited a comparable detection rate to the reference qPCR assay, except for the samples collected at 5 dpi, where it detected the virus in only 4 of 5 samples. On the other hand, the LFIA gave positive results sporadically at 4, 6, and 9 dpi. The maximum detection rate for LFIA was observed at 6 dpi, when it identified the virus in 3 of 5 positive samples (60%) (Fig. 3B).

Similar to the whole-blood samples, we analyzed 82 oral swabs, including 35 collected before and 47 collected after inoculation. Thirty-two swabs tested positive, and 50 tested negative by the reference qPCR assay (Tables 3 and 4). Both pen-side tests accurately identified all 50 negative samples that tested negative by the reference qPCR assay. The portable qPCR test identified 30 out of 32 (93.8%) samples that tested positive by the reference qPCR assay (Table 3). In contrast, the LFIA detected only six out of 32 (18.8%) samples that tested positive by the reference qPCR assay (Table 4). Notably, we did not observe any correlation between the Ct values and the performance of the LFIA. Interestingly, the six oral swabs that tested positive by the LFIA exhibited higher Ct values than those that tested negative by the LFIA (Supplementary Table S2).

**Table 3:** Comparison of portable qPCR and reference qPCR test results for oral swabs

Portable qPCR	Reference qPCR		Total
	Positive	Negative	
Positive	30	0	30
Negative	2	50	52
Total	32	50	82

Sensitivity: 93.8% Specificity: 100%

**Table 4:** Comparison of LFIA and reference qPCR test results for oral swabs

LFIA	Reference qPCR		Total
	Positive	Negative	
Positive	6	0	6
Negative	26	50	76
Total	32	50	82

Sensitivity: 18.8% Specificity: 100%

## Discussion

Whole blood samples are currently the recommended sample type for ASFV diagnosis in live animals [16]. Here, we investigated the potential utility of oral swabs as an alternative sample matrix. In this study, we consistently detected viral genomic DNA in all whole blood samples collected from 2 dpi and in all oral swabs collected from 4 dpi, using a reference qPCR assay. Thus, the data indicate that ASFV appeared in blood earlier than in oral secretions. Additionally, blood samples contained higher viral loads than oral swabs, evidenced by lower Ct values in the reference qPCR tests for blood samples than for oral swabs (Supplementary Fig. S1). Thus, our results are consistent with those of previous studies [11]. Despite these differences, oral swabs can still be a valuable sample type for ASFV diagnosis because, starting from 4 dpi, all pigs consistently had viral genomic DNA in their oral swabs.

Both the LFIA and portable qPCR test successfully detected infection in pigs before the onset of clinical signs when testing whole blood samples. We observed a significant difference in sensitivity between these two pen-side tests. The portable qPCR test detected viral DNA in 40 (97.6%) of the 41 whole blood samples that tested positive by the reference qPCR assay, whereas the LFIA detected viral antigens in only 27 PCR-positive samples (65.9%). The lower sensitivity of the LFIA compared to the portable qPCR test observed in this study aligns with findings in COVID-19 cases, where LFIA for detecting viral antigens usually exhibits less sensitivity than PCR tests [3, 6]. This discrepancy arises because real-time PCR exponentially amplifies viral genomes, allowing trace amounts of the viral genome to be detected.

The LFIA’s sensitivity is greatly affected by the viral load present in the test sample. When testing with tissue culture virus, the LFIA showed a detection limit of 10^4^ plaque-forming units per mL [23]. When testing whole blood samples collected from experimentally infected or exposed pigs, the LFIA demonstrated a higher false negative rate for samples with Ct values above 30 [23]. However, it was observed that a high proportion of the whole blood samples that tested negative by the LFIA test also exhibited Ct values below 30. Specifically, out of 17 whole blood samples collected from the field that yielded negative results by the LFIA, 10 (58.8%) had Ct values below 30. Among these 17 false-negative samples, three had Ct values ranging from 21 to 22 [23]. Similarly, it has been noted that the LFIA’s sensitivity decreases significantly when used to test blood collected from boar carcasses, despite these samples showing notably low Ct values [8]. In this study, the LFIA failed to detect the virus in seven blood samples collected at 7 dpi or later despite their low Ct values (Supplementary Table S1). Thus, it is evident that the LFIA’s sensitivity is influenced not only by the viral load but also by other unknown factors in the samples.

The portable qPCR device demonstrated high sensitivity when testing oral swabs, while the LFIA exhibited low sensitivity with this sample type. The LFIA was initially designed for use with EDTA-blood [23]. Interestingly, it was reported recently that the LFIA demonstrated significantly higher sensitivity when testing serum samples than when testing EDTA-treated blood [19], despite serum containing lower viral loads than whole blood. This observation suggests that LFIA could potentially be used for testing with different sample matrices. In this study, we observed a very low sensitivity of the LFIA when testing oral swabs. Among 32 samples that tested positive using the reference qPCR assay, the LFIA detected only six (18.8%). Hence, oral swabs are considered unsuitable for LFIA testing.

Due to its lower sensitivity, LFIA test results must be interpreted with caution, as false negative results could hinder disease control and prevention measures. The LFIA might be more valuable for herd diagnosis rather than individual animal diagnosis. In this context, the lower test sensitivity could be compensated by testing a larger number of samples, thereby increasing the likelihood of identifying an infected pig. Additionally, when testing pigs without clinical signs that receive a negative LFIA test result, it is advisable to repeat the test 2 or 3 days later, because the LFIA failed to detect the virus in samples collected very early after inoculation. The strategy of retesting to enhance the diagnostic sensitivity of antigen tests has been documented in the case of COVID-19 [6].

In summary, both the portable qPCR test and LFIA successfully detected ASFV before the onset of clinical signs, especially when testing whole blood samples. These tests could be important tools for effective disease control and management, allowing immediate implementation of biosecurity measures to mitigate the risk of virus transmission to other herds.

### Electronic Supplementary Material

Below is the link to the electronic supplementary material

Supplementary Material 1

## Data Availability

All data supporting the main findings of this study are included in the published article.
