# Supplementary material for: Evaluation of the diagnostic sensitivity and specificity of two pen-side tests for detecting African swine fever virus in experimentally infected pigs
Source: Arch Virol. 2024 Jul 30;169(8):170. doi: 10.1007/s00705-024-06098-0 (PMC11289199; doi:10.1007/s00705-024-06098-0)
Supplement: Supplementary file 1 — Supplementary Material 1 [file 705_2024_6098_MOESM1_ESM.docx]

# Supplementary Data

Figure S1: Comparison of Ct values obtained from the reference qPCR when testing whole blood and oral swabs. The horizontal dashed line at a Ct value 38 indicates the assay cut-off. Data were analyzed by the paired Student’s T test. **** p<0.0001

**Table S1: Whole blood samples tested positive by the reference qPCR**

| Sample ID | Pig ID | dpi | Reference qPCR | Portable qPCR | LFIA |
| --- | --- | --- | --- | --- | --- |
| WB1 | 2 | 2 | 32.27 | 35.2 | Negative |
| WB2 | 4 | 2 | 29.87 | 32.8 | Negative |
| WB3 | 6 | 2 | 36.5 | 40 | Negative |
| WB4 | 8 | 2 | 34.99 | 37.5 | Negative |
| WB5 | 10 | 2 | 33.94 | 35.4 | Negative |
| WB6 | 1 | 3 | 31.55 | 32.3 | Negative |
| WB7 | 5 | 3 | 26.17 | 29.8 | Negative |
| WB8 | 7 | 3 | 25.22 | 27.7 | Positive |
| WB9 | 9 | 3 | 22.81 | 25.4 | Positive |
| WB10 | 2 | 4 | 27.06 | 24.7 | Positive |
| WB11 | 4 | 4 | 20.41 | 22.3 | Positive |
| WB12 | 6 | 4 | 22.18 | 24.7 | Positive |
| WB13 | 8 | 4 | 22.02 | 23.4 | Positive |
| WB14 | 10 | 4 | 21.22 | 23.2 | Positive |
| WB15 | 1 | 5 | 22.31 | 25.0 | Positive |
| WB16 | 3 | 5 | 25.07 | 29.3 | Positive |
| WB17 | 5 | 5 | 20.66 | 23.8 | Positive |
| WB18 | 7 | 5 | 21.34 | 22.9 | Positive |
| WB19 | 9 | 5 | 21.07 | 23.1 | Positive |
| WB20 | 2 | 6 | 20.7 | 23.7 | Positive |
| WB21 | 4 | 6 | 21.64 | 23.3 | Positive |
| WB22 | 6 | 6 | 21.51 | 23.8 | Positive |
| WB23 | 8 | 6 | 22.14 | 23.0 | Positive |
| WB24 | 10 | 6 | 21.08 | 22.6 | Positive |
| WB25 | 1 | 7 | 22.85 | 25.6 | Positive |
| WB26 | 3 | 7 | 24.8 | 29.9 | Negative |
| WB27 | 5 | 7 | 20.04 | 24.5 | Positive |
| WB28 | 7 | 7 | 21.35 | 24.7 | Positive |
| WB29 | 9 | 7 | 20.95 | 24.3 | Positive |
| WB30 | 2 | 8 | 21.18 | 25.4 | Negative |
| WB31 | 6 | 8 | 21.23 | 24.4 | Positive |
| WB32 | 8 | 8 | 20.57 | 24.4 | Positive |
| WB33 | 10 | 8 | 19.08 | 21.9 | Positive |
| WB34 | 1 | 9 | 21.65 | 25.8 | Positive |
| WB35 | 3 | 9 | 26.83 | 29.9 | Negative |
| WB36 | 5 | 9 | 20.59 | 24.3 | Negative |
| WB37 | 7 | 9 | 22.01 | 24.6 | Negative |
| WB38 | 9 | 9 | 22.03 | 25.2 | Negative |
| WB39 | 2 | 10 | 24.07 | 28.3 | Positive |
| WB40 | 6 | 10 | 23.92 | 27.7 | Negative |
| WB41 | 8 | 10 | 22.45 | 27.8 | Positive |

**Table S2: Oral swabs tested positive by the reference qPCR**

| Sample ID | Pig ID | dpi | Reference qPCR | Portable qPCR | LFIA |
| --- | --- | --- | --- | --- | --- |
| OSw1 | 5 | 3 | 37.74 | 40 | Negative |
| OSw2 | 2 | 4 | 24.11 | 29.1 | Negative |
| OSw3 | 4 | 4 | 26.48 | 28.7 | Negative |
| OSw4 | 6 | 4 | 30.84 | 31.8 | Negative |
| OSw5 | 8 | 4 | 28.19 | 31.2 | Positive |
| OSw6 | 10 | 4 | 26.60 | 28.7 | Negative |
| OSw7 | 1 | 5 | 32.33 | 33.3 | Negative |
| OSw8 | 3 | 5 | 31.30 | 40 | Negative |
| OSw9 | 5 | 5 | 23.73 | 27.5 | Negative |
| OSw10 | 7 | 5 | 24.48 | 28.5 | Negative |
| OSw11 | 9 | 5 | 26.52 | 30.2 | Negative |
| OSw12 | 2 | 6 | 24.58 | 30.4 | Negative |
| OSw13 | 4 | 6 | 27.79 | 32.3 | Positive |
| OSw14 | 6 | 6 | 26.40 | 32.3 | Negative |
| OSw15 | 8 | 6 | 24.48 | 25.7 | Positive |
| OSw16 | 10 | 6 | 26.19 | 27.3 | Positive |
| OSw17 | 1 | 7 | 30.03 | 30.9 | Negative |
| OSw17 | 3 | 7 | 33.73 | 35.3 | Negative |
| OSw19 | 5 | 7 | 26.66 | 30.2 | Negative |
| OSw20 | 7 | 7 | 28.06 | 28.9 | Negative |
| OSw21 | 9 | 7 | 26.61 | 30.3 | Negative |
| OSw22 | 2 | 8 | 27.22 | 29.2 | Negative |
| OSw23 | 6 | 8 | 28.24 | 29.7 | Negative |
| OSw24 | 8 | 8 | 31.19 | 29.8 | Negative |
| OSw25 | 10 | 8 | 29.84 | 32.9 | Negative |
| OSw26 | 1 | 9 | 30.00 | 32.0 | Negative |
| OSw27 | 5 | 9 | 29.97 | 35.0 | Negative |
| OSw28 | 7 | 9 | 30.12 | 35.5 | Positive |
| OSw29 | 9 | 9 | 29.05 | 36.4 | Positive |
| OSw30 | 2 | 10 | 27.18 | 31.2 | Negative |
| OSw31 | 6 | 10 | 25.05 | 31.0 | Negative |
| OSw32 | 8 | 10 | 29.31 | 33.1 | Negative |
